# *Cytomegalovirus* Infection Impairs Immunosuppressive and Antimicrobial Effector Functions of Human Multipotent Mesenchymal Stromal Cells

**DOI:** 10.1155/2014/898630

**Published:** 2014-03-23

**Authors:** Roland Meisel, Kathrin Heseler, Julia Nau, Silvia Kathrin Schmidt, Margret Leineweber, Sabine Pudelko, Johannes Wenning, Albert Zimmermann, Hartmut Hengel, Christian Sinzger, Özer Degistirici, Rüdiger Volker Sorg, Walter Däubener

**Affiliations:** ^1^Clinic for Pediatric Oncology, Hematology and Clinical Immunology, Center for Child and Adolescent Health, Medical Faculty, Heinrich-Heine-University, Moorenstraße 5, 40225 Düsseldorf, Germany; ^2^Institute of Medical Microbiology and Hospital Hygiene, Medical Faculty, Heinrich-Heine-University, Universitaetsstraße 1, 40225 Düsseldorf, Germany; ^3^Institute for Virology, Medical Faculty, Heinrich-Heine-University, Universitaetsstraße 1, 40225 Düsseldorf, Germany; ^4^Institute for Virology, University Medical Centre Freiburg, Hermann Herder Straße 11, 79104 Freiburg, Germany; ^5^Institute for Virology, University of Ulm, Albert Einstein Allee, 89081 Ulm, Germany; ^6^Institute for Transplantation Diagnostics and Cell Therapeutics, Medical Faculty, Heinrich-Heine-University, Moorenstraße 5, 40225 Düsseldorf, Germany

## Abstract

Human mesenchymal stromal cells (MSC) possess immunosuppressive and antimicrobial effects that are partly mediated by the tryptophan-catabolizing enzyme indoleamine-2,3-dioxygenase (IDO). Therefore MSC represent a promising novel cellular immunosuppressant which has the potential to control steroid-refractory acute graft versus host disease (GvHD). In addition, MSC are capable of reducing the risk of infection in patients after haematopoietic stem cell transplantation (HST). Recent data indicate that signals from the microenvironment including those from microbes may modulate MSC effector functions. As *Cytomegalovirus* (CMV) represents a prominent pathogen in immunocompromised hosts, especially in patients following HST, we investigated the impact of CMV infection on MSC-mediated effects on the immune system. We demonstrate that CMV-infected MSC lose their cytokine-induced immunosuppressive capacity and are no longer able to restrict microbial growth. IDO expression is substantially impaired following CMV infection of MSC and this interaction critically depends on intact virus and the number of MSC as well as the viral load. Since overt CMV infection may undermine the clinical efficacy of MSC in the treatment of GvHD in transplant patients, we recommend that patients scheduled for MSC therapy should undergo thorough evaluation for an active CMV infection and receive CMV-directed antiviral therapy prior to the administration of MSC.

## 1. Introduction

Human multipotent mesenchymal stromal cells (MSC), known for their multilineage differentiation potential, possess pleiotropic immunosuppressive functions that are partly mediated by expression of the tryptophan-catabolizing enzyme indoleamine-2,3-dioxygenase (IDO) [1–4]. Upon stimulation with inflammatory cytokines, MSC exhibit broad-spectrum antimicrobial effector functions directed against various clinically relevant pathogens, and these effects are dependent on IDO and/or the antimicrobial peptide LL-37 [5, 6]. These dual immunosuppressive and antimicrobial properties render MSC a promising novel cellular immunosuppressant which is currently under intensive clinical investigation for various auto- and alloimmune diseases such as steroid-refractory graft versus host disease (GvHD) after allogeneic hematopoietic stem cell transplantation (HSCT), Crohn's disease, and multiple sclerosis [7–10]. Emerging data indicate that signals from the microenvironment including those induced by hypoxia [11, 12] or derived from microbes may critically affect IDO and consequently MSC effector functions [13–15]. As the* Cytomegalovirus* (CMV) represents a prominent pathogen in immunocompromised hosts in particular in patients suffering from GvHD after HSCT, we initiated studies investigating the impact of CMV infection on MSC-mediated effects. During coevolution with its specific host, human CMV has developed several immune evasion strategies [16–18]. For example, CMV has been reported to inhibit the upregulation of MHC class II antigens. Furthermore, it was found that CMV generally inhibits signalling via the IFN-*γ* receptor and that this is mediated via a reduced phosphorylation of STAT1 and an enhanced degradation of Jak1 [19–21].

Mesenchymal stromal cells and embryonic stem cells are able to inhibit T-cell responses and several mechanisms including the production of prostaglandins, of immunosuppressive cytokines [3], of arginase I [22], or of adenosine [23, 24] appear to be involved in this effect. In addition, we and others reported that the immunoregulatory effects of mesenchymal stromal cells are, at least in part, brought about by the induction of the tryptophan degrading enzyme indoleamine 2,3-dioxygenase [4].

We report here that CMV is a major negative regulator of IDO activity in human MSC, dramatically reducing their immunosuppressive and antimicrobial properties, thereby implicating that active CMV infections may undermine the clinical efficacy of MSC treatment.

## 2. Materials and Methods

### 2.1. Primary Cells

Human bone marrow-derived MSC were prepared, propagated, and characterized as previously described [5]. Bone marrow aspirates for the generation of MSC were obtained from healthy volunteer donors who had provided written informed consent; the study was conducted according to the Declaration of Helsinki principles and approved by the ethics committee of the Medical Faculty of the Heinrich-Heine-University, Düsseldorf, Germany.

### 2.2. Cell Lines and Reagents

OKT3 producing hybridoma cells were obtained from the American Type Culture Collection (Rockville, USA). Recombinant human IFN-*γ* was purchased from R&D Systems (Wiesbaden, Germany). L-Tryptophan, L-kynurenine, 1-L-methyl-tryptophan (1-MT), and Ehrlich's reagent were ordered from Sigma-Aldrich (Deisenhofen, Germany).

### 2.3. Human* Cytomegalovirus*

CMV strains AD169 and TB40E were kindly provided by C. Sinzger (Institute for Medical Virology, Ulm, Germany) and A. Zimmermann (Institute for Virology, Düsseldorf, Germany). Before infection of MSC, the virus-containing solution was thawed and diluted in tryptophan-free RPMI 1640 medium to reach a multiplicity of infection (MOI) of 0.1–10. In some experiments UV-inactivated CMV preparations were used. Viral replication was analysed using real-time PCR as described [5].

### 2.4. Kynurenine Assay

The enzymatic activity of IDO directly correlates with the concentration of kynurenine in supernatants of tissue culture cells and therefore, the measurement of kynurenine can be used to determine IDO activity [25]. MSC (2 × 10^4^ per well) were plated in 96-well flat-bottomed microtiter plates in IMDM containing 5% FCS and 0.6 mM L-tryptophan. The cultures were stimulated with IFN-*γ* at concentrations indicated in the respective experiments. The plates were incubated at 37°C and after 72 h 160 *μ*L were removed from each well and transferred to a 96-well V-bottomed plate. After the addition of 10 *μ*L 30% trichloroacetic acid to each well, the plates were incubated at 50°C for 30 minutes to hydrolyze N-formyl-kynurenine to kynurenine. After centrifugation for 10 min at 600 g, 100 *μ*L supernatant was transferred to 96-well flat-bottomed plates and 100 *μ*L 1.2% (w/v) 4-(dimethylamino) benzaldehyde (Ehrlich's reagent) in glacial acetic acid was added. After 10 minutes at room temperature, the extinction was determined at 492 nm with a microplate reader (Tecan, Crailsheim, Germany). Data are given as mean kynurenine content of triplicate cultures. In some experiments IDO was induced by coculturing MSC with OKT3-stimulated peripheral blood lymphocytes for three days. As a control 1-L-MT (1.5 mM) or a neutralizing anti-IFN-*γ* antibody (10 ng/mL) was added at the time point of MSC stimulation. In addition, IDO protein was detected in stimulated MSC using Western blot analysis as described [5].

### 2.5. T-Cell Proliferation Assay

1 × 10^5^ peripheral blood lymphocytes (PBL), obtained from heparinised blood of healthy donors after Ficoll purification, were stimulated with a monoclonal anti-CD3 antibody (OKT3, American Type Culture Collection, Rockville, USA) in the presence of different amounts of MSC as described [26]. In some experiments MSC (0.5–2 × 10^4^ per well) were infected with CMV and/or stimulated with IFN-*γ* at the start of the culture. After three days the cultures were pulsed with 0.2 *μ*Ci [^3^H] thymidine for 24 hours. T-cell proliferation was measured by [^3^H] thymidine incorporation using liquid scintillation spectrometry (1205 Betaplate, PerkinElmer, Jugesheim, Germany).

### 2.6. *Staphylococcus aureus*

MSC were infected with 10–100 cfu/well. Bacterial growth was monitored after further incubation of 16 h by measuring optical density at 620 nm [27].

### 2.7. Statistical Analysis

All data are given as mean ± SEM of at least 3 independent experiments and each experiment was performed in triplicate. Data of representative experiments, also performed in triplicate, were given as mean ± SD. For the comparison of different data Student's *t*-test for unpaired groups was used and the *P* value was calculated using GraphPad Prism software.

## 3. Results and Discussion

**Figure 1:**
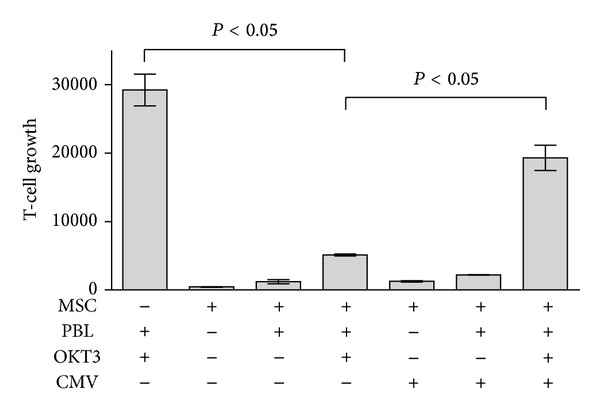
CMV infection abrogates the immunosuppressive effect of human MSC. Peripheral blood lymphocytes (PBL; 1 × 10^5^/well) were activated with a CD3-directed mAb (OKT3) and cultured in the presence or absence of MSC (2 × 10^4^/well) which were infected with CMV (MOI 5) or not. After three days T-cell proliferation was assessed using [^3^H] thymidine incorporation. Data are displayed as mean cpm ± SD of a representative experiment performed in triplicate.

**Figure 2:**
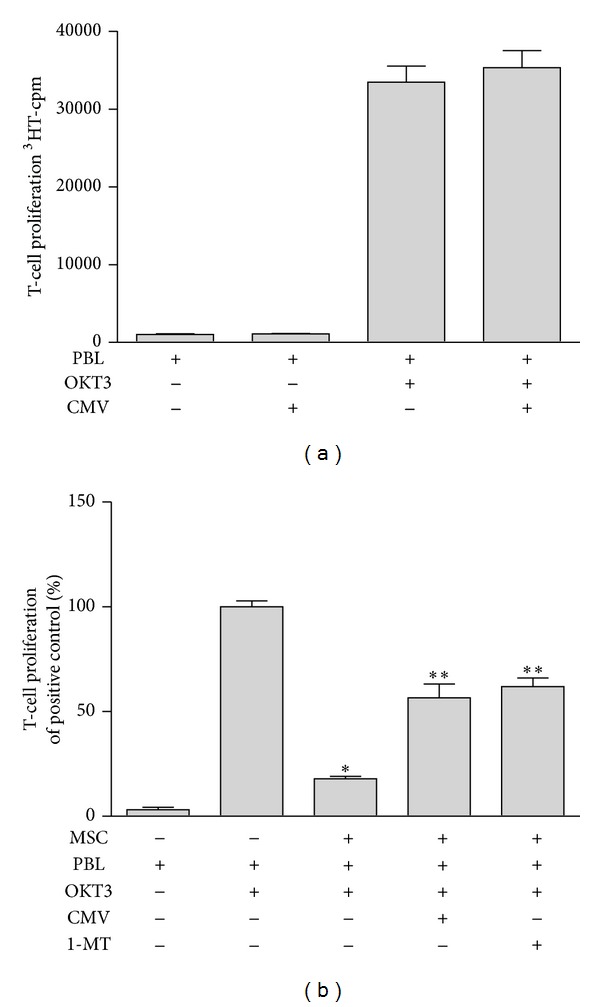
Influence of MSC and CMV on T-cell proliferation. (a) PBL were cultured in the absence or presence of CMV (corresponding to MOI 5) for three days and were stimulated with OKT3 or not. Data are given as mean cpm [^3^H] thymidine incorporation of four independent experiments with cells from different donors. (b) PBL were cultured in the presence of MSC (3 × 10^4^/well) and in the absence or presence of 1-MT (1.5 mM) or CMV (MOI 5). T-cell proliferation was determined as described above. Data are given as % of positive control (OKT3-activated PBL without MSC). The significant inhibition of T-cell proliferation by MSC is marked with one asterisk (*P* < 0.05); the significant antagonistic effect on T-cell inhibition by CMV or 1MT is marked with two asterisks (*P* < 0.05).

The profound T-cell inhibitory capacity of human multipotent mesenchymal stromal cells has raised much interest promting studies investigating MSC as a novel cellular immunosuppressant, in particular in steroid-refractory GvHD after HSCT. To explore the potential impact of CMV infections on the therapeutic efficacy of MSC in this setting, we set up a three-party cell culture system, in which OKT3-induced T-cell proliferation was assessed in the presence or absence of human MSC that were infected with CMV at a multiplicity of infection 5 (MOI 5) or left untreated. As shown in Figure 1 we found a profound T-cell inhibitory effect of MSC, which was partially but significantly reversed in the presence of CMV. This effect, however, did not rely on CMV-directed T-cell responses as CMV-infected MSC did not induce any T-cell proliferation in the absence of OKT3 stimulation (Figure 1). In addition, T cells from our donors did not proliferate in the presence of CMV and CMV did not significantly alter the T-cell response after stimulation with anti-CD3 antibodies as shown in Figure 2(a). We have previously shown that the induction of IDO is, at least in part, responsible for the reduced T-cell proliferation induced by MSC [4]. To prove this finding we used the IDO inhibitor 1-L-methyl-tryptophan (1MT). As shown in Figure 2(b) human MSCs inhibited T-cell responses and this inhibitory effect could be abrogated by the addition of CMV or of 1-MT.

**Figure 3:**
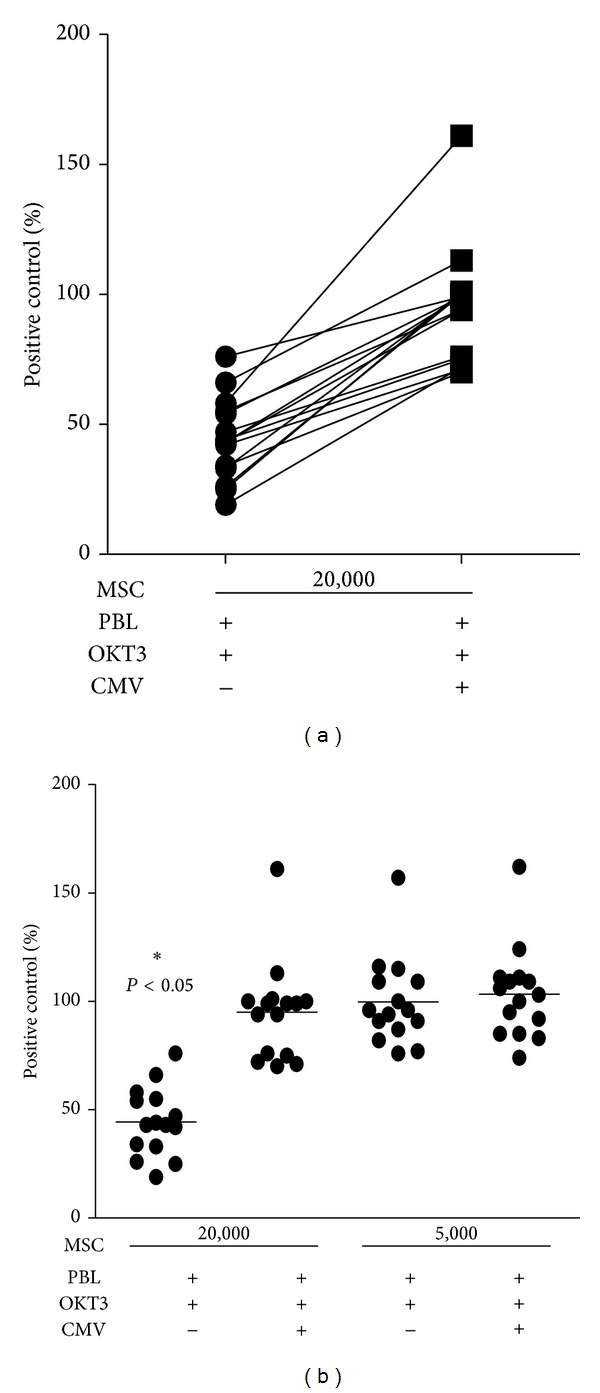
Dose dependency of MSC-mediated effects on T-cell proliferation. Different amounts of MSC ((a) 2 × 10^4^/well and (b) 2 × 10^4^ or 5 × 10^3^/well), either CMV infected (MOI 5) or not, were cocultured with OKT3-activated PBL. Thereafter, T-cell proliferation was determined as described above. Data are given as % of positive control, that is, OKT3-activated PBL without MSC. Each dot represents a single data point from a total of 15 individual experiments, each performed in triplicate. A significant inhibition of T-cell proliferation by MSC is marked with asterisks.

To substantiate these experimental findings, we performed a series of 15 subsequent experiments employing various numbers of CMV-infected MSC. We found that 2 × 10^4^ MSC provided substantial suppression of T-cell proliferation and the addition of CMV consistently antagonizes this T-cell inhibitory effect. In all 15 experiments performed an inhibitory effect of CMV on MSC-mediated T-cell suppression was observed; however, the magnitude of this effect varied (Figure 3(a)). In contrast, 5 × 10^3^ MSC were unable to restrict T-cell proliferation and, under these conditions, the same amount of CMV that was employed in the experiments shown in Figure 3(b) did not have any impact on T cells, thus ruling out an unspecific effect of CMV. Taken together, this data demonstrates that CMV infection of human MSC substantially impedes their T-cell inhibitory effector function.

**Figure 4:**
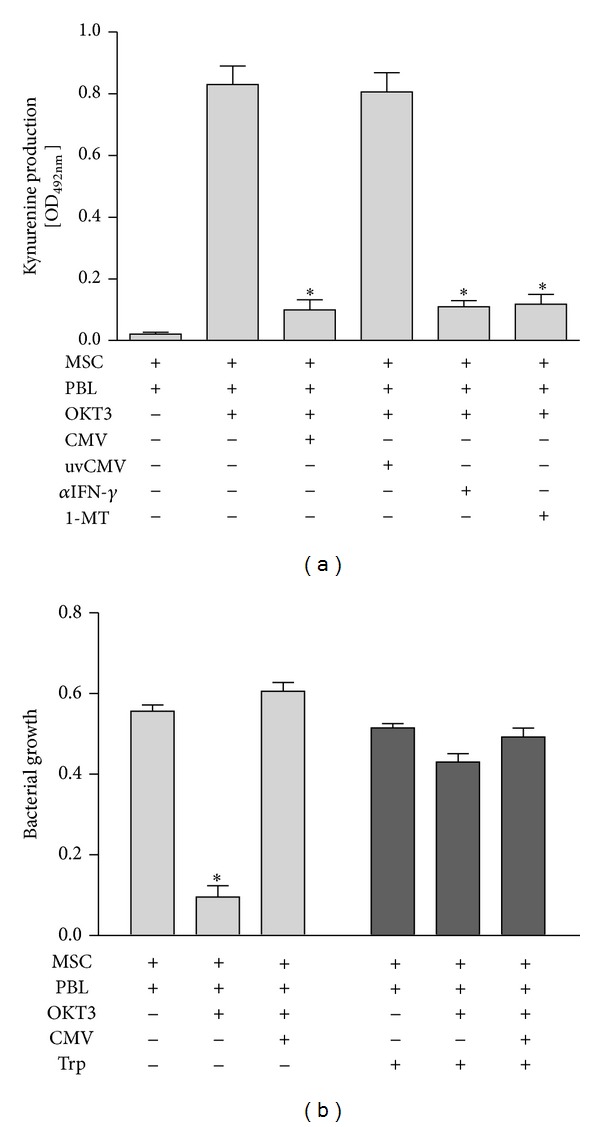
CMV blocks IDO activity and IDO-mediated antimicrobial effects in human MSC. (a) PBL, stimulated with OKT3, were cocultured with MSC in the presence or absence of CMV. As controls UV-inactivated CMV (uvCMV), the IDO-specific inhibitor 1-L-methyl-tryptophan (1-MT; 1.5 mM), or a neutralising anti-IFN-*γ* antibody (*α*IFN-*γ*; 10 ng/mL) was used. After three days IDO activity was determined and is presented as mean kynurenine content ± SEM of three independent experiments, each done in triplicate. (b) PBL (1 × 10^5^/well), stimulated with CD3-directed mAB OKT3, were cocultured with MSC (3 × 10^4^/well) in the absence or presence of CMV (MOI 5). After three days cultures were infected with* S. aureus* (10–100 cfu/well) and bacterial growth was determined photometrically. As a control, cultures were supplemented with L-tryptophan (Trp; 0.6 mM) at the time point of bacterial infection. Data are given as mean OD_(620 nm)_  ± SEM of three experiments, each done in triplicate. Significant differences (*P* < 0.05) as compared to the positive control are marked by asterisks.

In previous studies we and others have identified a significant role of the IFN-*γ*-inducible tryptophan-catabolizing enzyme indoleamine-2,3-dioxygenase (IDO) in MSC-mediated T-cell inhibition [4, 28]. In additional studies, the role of IDO in tolerance induction was demonstrated in* in vivo* studies using IDO deficient animals [29]. Based on these findings we went on to assess the impact of CMV infection on cytokine-induced IDO activity of human MCS. As shown in Figure 4(a), we observed a substantial IDO-mediated kynurenine production when MSC were cultured in the presence of OKT3-activated T cells. As expected, IDO activity induced in MSC by activated T cells could be blocked by neutralising antibodies directed against IFN-*γ* as well as by the IDO-specific inhibitor 1-L-methyl-tryptophan. However, it is of particular interest that CMV-infected MSC were unable to express IDO activity in the presence of activated T cells, while UV-inactivated CMV preparations had no impact on IDO activity. Thus, an infection with replication-competent CMV substantially impairs IFN-*γ*-induced IDO expression in human MSC.

In addition to their immunosuppressive capacity, MSC have recently been shown to possess cell-autonomous antimicrobial effects directed against various clinically relevant pathogens [5, 6, 15]. We therefore proceeded to analyse the potential impact of CMV on MSC-mediated antibacterial effects induced by cytokines released from OKT3-stimulated T cells. As shown in Figure 4(b), MSC cocultured with activated T cells are able to restrict bacterial growth and CMV infection of MSC abrogated their antimicrobial effect against* Staphylococcus aureus*. The functional relevance of IDO-mediated antibacterial effects was confirmed by demonstrating that addition of excess amounts of tryptophan completely abolished the antimicrobial effector function of MSC (Figure 4(b)).

**Figure 5:**
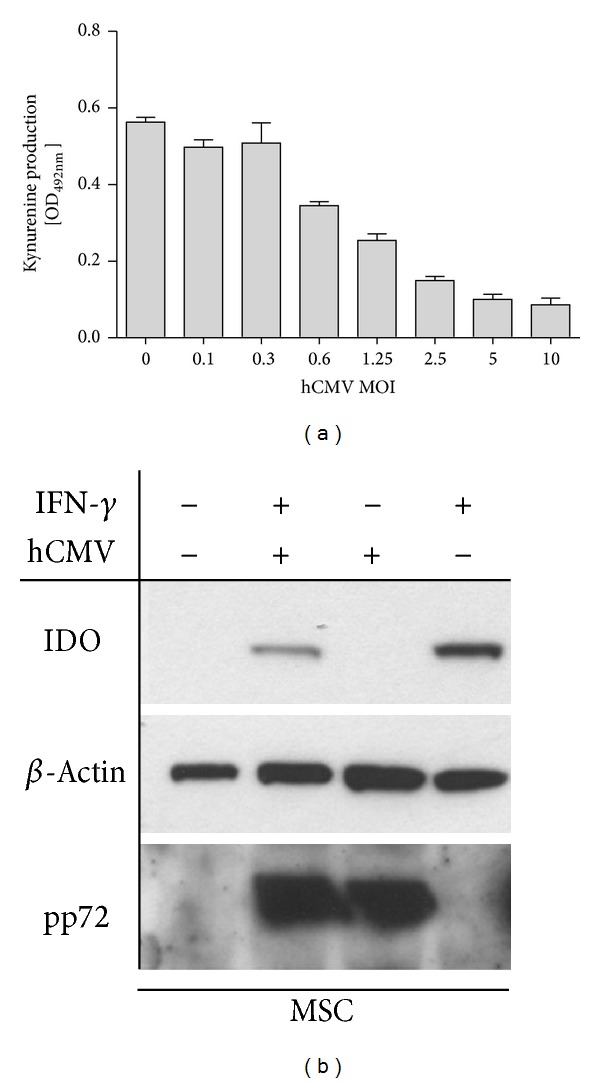
CMV inhibits IDO induction by recombinant IFN-*γ*. (a) MSC (2 × 10^4^/well) which were infected with various amounts of CMV (MOI 0.1–10) were stimulated with IFN-*γ* (300 U/mL). After three days IDO activity was determined and is presented as mean kynurenine content ± SEM of three independent experiments, each done in triplicate. (b) MSC (1.5 × 10^6^/flask) were stimulated with IFN-*γ* (600 U/mL) in the absence or presence of CMV (MOI 5). Cells were harvested after 24 h and IDO protein was detected in Western blot analysis. *β*-Actin was utilized as a protein loading control, while the viral pp72 protein served as an infection control.

In additional experiments we analysed CMV-mediated inhibition of IFN-*γ* induced IDO activity in human MSC in more detail. We found that CMV infection impairs cytokine-induced IDO activity of human MSC in a dose-dependent manner with significant inhibition observed at CMV doses as low as a MOI of 0.6 and with a maximum effect at a MOI of 5 (Figure 5(a)). Furthermore, Western blot analysis, depicted in Figure 5(b), showed that CMV infection of MSC results in a reduced expression of IDO protein.

In our present work we describe for the first time that a CMV infection critically impairs the immunosuppressive and antimicrobial effector functions of human MSC possibly via an interaction with the IFN-*γ*-induced IDO pathway. We demonstrate that this interaction between CMV and IDO-mediated effector functions of stromal cells critically depends on intact virus as well as the number of host cells and virus employed.

We have recently reported that human fibroblasts lose their immunosuppressive capacity after a CMV infection and that this finding might be relevant for host-versus-graft reaction disease triggered by CMV infection/reactivation after solid organ transplantation [26]. Here we show that a similar CMV-mediated effect on MSC might influence the clinical effectivity of these cells as immunosuppressant.

We are aware of the fact that our observations are derived from* in vitro* cell culture experiments. In the* in vivo* situation an immunosuppressive therapy, necessary in transplant patients, might result in an inhibition of IFN-*γ* production and therefore inhibit IDO induction [30]. Immunosuppressive substances such as glucocorticoides at high concentrations are able to enhance IDO activity [31, 32]. Furthermore the broad organ tropism, different cell types, and differences in cell tropism might have an impact on CMV-mediated inhibition of IDO activation [33]. However, based on the species specificity of CMV infection as well as IDO expression [5, 34], murine* in vivo* experiments including humanized xenograft models will unfortunately not be informative with regard to the* in vivo* impact of a CMV infection on MSC-mediated immunosuppressive and antimicrobial effects.

We put a particular focus on MSC in our study as these cells represent a promising novel therapeutic approach for a variety of clinical applications ranging from tissue engineering and regenerative medicine to the treatment of auto- and alloimmune diseases refractory to conventional pharmacologic immunosuppression [35, 36]. One might speculate that the inhibitory interaction of CMV with IDO-mediated immunosuppressive effects may explain the consistent clinical observation that CMV infection frequently triggers GvHD following hematopoietic stem cell transplantation [37].

The findings presented here may have implications for transplantation medicine and the future clinical use of MSC. We found that MSC and fibroblasts are permissive for CMV infection and are able to inhibit viral growth due to IDO-dependent mechanisms [5]. Both cell types are also sensitive to CMV-mediated immune escape mechanisms [26]. We suggest that the balance between IFN-*γ* dose-dependent IDO induction and CMV dose-dependent IDO inhibition might influence the clinical outcome of organ or stem cell transplantation in CMV-infected patients. We are aware that the magnitude of virus load and of IFN-*γ* concentrations in virus plaques within infected human tissues is unknown. However we recommend that patients scheduled for MSC therapy should undergo thorough evaluation for an active CMV infection and receive CMV-directed antiviral therapy prior to administration of MSC, if appropriate, since overt CMV infection of MSC recipients may undermine the clinical efficacy of the MSC treatment.

Thus, strategies that aim at restoring IDO expression in CMV-infected MSC may prove beneficial in certain clinical scenarios. Further studies characterizing the distinct signalling pathways that are targeted by CMV and result in modulation of IDO expression in MSC are necessary to identify molecular targets for therapeutic intervention.
